# Food safety and nutrition for low-income urbanites: exploring a social justice dilemma in consumption policy

**DOI:** 10.1177/0956247819858019

**Published:** 2019-10

**Authors:** Sigrid Wertheim-Heck, Jessica Evelyn Raneri, Peter Oosterveer

**Affiliations:** 1 Sigrid Wertheim-Heck is a professor of Food and Healthy Living at Aeres University of Applied Sciences and a senior research fellow at the Environmental Policy Group at Wageningen University, both in the Netherlands. Her interest in global urban food security informs her research agenda on the relationship among metropolitan development, food provisioning and food consumption. With a background in consumption sociology, her main areas of interest include everyday consumption practices and equitable access to sustainable, safe and healthy foods; 2 Jessica Raneri is a nutrition research specialist at Bioversity International. She is involved in designing and implementing “Agricultural Biodiversity, Nutrition and Dietary” assessments using participatory, qualitative and quantitative methods. She leads a project in Vietnam designed to improve dietary diversity through an integrated systems perspective. She also supports sustainable diet activities and believes that it is crucial to understand how biodiversity can be utilized to improve the sustainability of food systems and quality of diets; 3 Peter Oosterveer is a professor at the Environmental Policy Group at Wageningen University, the Netherlands. His research interests are in global public and private food governance arrangements and innovative institutional developments in sustainable food production and consumption, in particular labelling and certification practices in global supply chains. Furthermore, he is studying food consumption practices from a sociological perspective and is particularly interested in how consumers access sufficient, sustainable and healthy food, including the role of retail

**Keywords:** food access, food retail environment, Hanoi, healthy diet, social equity, urbanization, Vietnam

## Abstract

Equitable access to healthy food is a critical challenge in urban Asia. Food safety governance promotes modern supermarkets over more traditional markets, but supermarkets are associated with unequal access to food. This study investigates how retail policies driven by food safety impact the diets of the urban poor in Hanoi, Vietnam. We do this by linking food retail infrastructures with the food shopping practices and measured dietary intake of 400 women. Our results reveal sub-optimal dietary diversity and reliance on foods sourced through traditional markets, which do not provide formal food safety guarantees. Modern channels supply formal food safety guarantees, but are mainly frequented for purchasing ultra-processed foods. The paper uncovers a conflicting duality governing food security and suggests that the public responsibility for ensuring access of the poor to nutritious and safe foods requires a more diverse retail policy approach.

## I Introduction

*“Of course, it is important for me to eat nutritious foods, but what is the point when they are not safe for consumption?”*^(1)^

This quote illustrates a prevalent dilemma in food health behaviour among urban low-income consumers in Asia. Over the past few decades there have been two notable shifts in the debate on food security. First, the focus has moved from quantity (mainly sufficient food energy) to the inclusion of quality in terms of safety, nutrition and (cultural) preferences.^(2)^ Second, where food security and nutrition have long been a primarily rural agenda, rapidly progressing urbanization and food system transitions now pose urgent challenges for sustainable, safe and nutritious food provisioning: food security has entered the urban agenda.^(3)^ This is most prominent in emerging economies of the global South, particularly Asia, where the rapid rate of urbanization results in a simultaneous rise in non-communicable diseases (NCDs)^(4)^ and urbanization of both poverty^(5)^ and malnutrition.^(6)^ A complicating challenge is food safety, a prominent and mounting issue for Asian emerging economies.^(7)^

Food safety concerns are impacting food retailing policies. Over recent decades, economic development, accelerated by the influx of foreign direct investment (FDI), has resulted in the supermarketization of food retail systems across Southeast Asia (SEA). This stimulates modern supermarket development at the expense of traditional food vending structures such as informal vending and wet markets. (Wet markets are open-air covered market spaces for fresh food, commonly found in Asian countries, in which wet refers to the wet floors due to the abundant use of water.)^(8)^ This supermarketization is encouraged by authorities based on the expectation that the formalization and modernization of retailing will contribute to improved hygiene practices and food safety through the implementation of private standards.^(9)^

The proliferation of modern retail outlets is, however, also associated with food access inequality. While policies that create incentives for private investments in supermarkets are partly framed as food safety efforts, they also contribute to broader processes of gentrification.^(10)^ This leads to transformations in the local food environment of inner-city neighbourhoods, including those inhabited by lower-income populations. Supermarket development primarily targets middle- and higher-income areas, with low-income neighbourhoods essentially becoming “supermarket deserts”. Furthermore, in Asia the replacement of formal wet markets by supermarkets has been demonstrated to exclude lower-income populations, as supermarkets are unaffordable, unfamiliar, or unwelcoming for them.^(11)^ Inadvertently, the basic human right of access to safe and healthy food for all is being challenged. There is a knowledge gap on how, and to what extent, transformations in the retail food environment have practical implications for food choice and dietary intake among low-income consumers.

Ensuring food security for urban low-income groups is among the toughest challenges confronting policymakers globally today. It is well recognized that lower-income groups in urban Asia are food and nutrition-insecure. Micronutrient deficiencies (especially iron and vitamin A) and the nutrition transition (increasing rates of overweight and obesity) are prevalent problems, requiring food-based strategies in response.^(12)^ Food security issues are different for low-income urban populations than for those living in rural areas. In urban areas, informal ties and reciprocities are weaker (weaker informal safety nets) and urban dwellers are more limited in terms of the self-sufficiency that comes with home production. Because low-income consumers in urban areas tend to buy a larger share of their food than rural dwellers, their dietary quality is more dependent on rising and volatile food price levels and changes in the retail environment. Research has demonstrated how the effect of income on diet is modified by the food environment, elucidating how food and nutrition insecurity may be attributed to food provision interventions.^(13)^ Food security entails access to safe food, but urban populations in Asia are increasingly confronted with serious public health concerns regarding the agrochemical and bacteriological safety of the most commonly consumed fresh foods: fruits and vegetables, meats and fish products.^(14)^ Thus, it is important to uncover the ways in which food environment transformations driven by food safety impact the diets of low-income groups.

Food and nutrition security of the growing number of low-income citizens depends on decisions for food system transformation made in urban planning. However, particularly for emerging economies, there is no well-developed evidence-based policy on how to adequately accommodate the needs of low-income groups. In Asia, there is a critical knowledge gap on the implications of food safety policies for the everyday diets of low-income consumers. Building on the case of Hanoi, this paper aims to understand how the food retail environment impacts food choice and dietary intake of low-income consumers, and to evaluate whether and in what way retail provisioning policies driven by food safety jeopardize the diets of food-vulnerable low-income consumers.

This paper continues with an introduction to the case of Hanoi (Section II), followed by a brief overview in Section III of the debate on whether food environments affect the diet quality of low-income consumers. Section IV presents the methods applied, and the results are presented in Section V. After the discussion in Section VI, we conclude with a summary of our findings and argue for the need to consider actual shopping practices when considering transformations of the (urban) food environment.

## II Hanoi, Vietnam: A Case in Point

*“People should buy food from supermarkets, malls or reputed shops to ensure food safety and hygiene.”*^(15)^

Our research focuses on Hanoi, the capital of Vietnam, which serves as an illustration for comparable Asian contexts regarding the food and nutrition security implications of rapid urban population growth, food system transformations and subsequent nutrition transitions. In Vietnam, a country listed among the world’s fastest-growing economies, the national urban population is expected to increase. Specifically, the population of Hanoi is projected to grow from about 7.5 million in 2015 to 9 million in 2030 and over 10 million in 2050.^(16)^ More than 40 per cent of Hanoi’s population currently lives below the poverty line of US$ 5/person/day.^(17)^ Previous research in Hanoi demonstrated how retail modernization policies lead to the exclusion of low-income groups, demonstrating that not all urban dwellers are being equally served.^(18)^ These policies aim to reduce the presence of wet markets, thereby depriving low-income groups of their traditional access points for fresh foods. This might, unintentionally, negatively impact their food choices and dietary intake. Traditional foods, including nutrient-rich dark green leafy vegetables, sesame and tofu, are becoming less prominent in the Hanoi diet, yet many of them have an important role in a healthy diet.^(19)^

Nevertheless, in preparing for the expected urban expansion, Hanoi authorities developed a master plan to transform Hanoi into a modern metropolis, including a transformation of the local food environment.^(20)^ In particular, retail modernization is emphasized in this plan.^(21)^ The Vietnamese government, and specifically the Hanoi People’s Committee (HPC), strives to reduce the provision of fresh foods via wet markets and informal street vending while it stimulates the development of supermarkets.^(22)^ Vietnamese policymakers actively restrict traditional retail structures, with plans aiming to reduce wet markets from the 67 permanent inner-city markets present in 2010 to 14 in 2020,^(23)^ while at the same time aiming to increase the number of supermarkets from around 60 in 2014 to 1,000 by 2025.^(24)^

The policy interventions to reduce the role of traditional markets in food retailing include: (i) restriction of the construction of new traditional markets; (ii) upgrading and renovation of markets; and (iii) transformation of markets into supermarkets. The development of new markets will be restricted in the sense that they are not included in the urban planning of new suburban residential areas. Policy interventions aimed at stimulating modern retail development include: (i) the development of supermarkets in office buildings and apartment complexes; (ii) suburban supermarket development; (iii) chain convenience store development; and (iv) establishment of supermarkets in department stores. Besides these policies aimed at the transformation of the formal food retail system, the modernization includes a more active repression of informal street vending practices, which range from ambulant vending to informally organized street markets.

These policies are reinforced by serious public health concerns regarding the agrochemical and bacteriological safety of the most commonly consumed fresh foods,^(25)^ since supermarkets are expected to implement private food safety management systems and maintain food hygiene standards.^(26)^ Moreover, by national regulation, all vegetables entering modern retail outlets are required to carry certificates issued by the Vietnamese authorities attesting that the vegetables have been produced in accordance with national regulations on safe vegetable production.^(27)^

Food safety is both a real problem and a perceived problem. Over the last decade in Vietnam, there has been an alarming increase in the inappropriate use of chemicals in agriculture.^(28)^ This has resulted in a stream of food safety incidents, which are widely covered in the public media. Consequently, Vietnamese consumers are anxious about the safety of the foods they consume on a daily basis. Although bacterial contamination is reported to be an important cause of foodborne disease,^(29)^ consumers generally believe they are able to avoid or minimize those risks, while they feel they lack the appropriate means to control the risks from agrochemicals.^(30)^ As a consequence, they are particularly concerned with chemical food hazards. These were initially primarily related to fresh vegetables and fruits, and more recently have extended increasingly to meat.^(31)^ The expectation is that the ubiquitous and urgent food safety concerns will drive the adoption of new and modern retail formats in the everyday lives of consumers.^(32)^ Still, in developing countries, even modern retailers are confronted with challenges in securing food safety, such as dealing with the fragmented supply chain.^(33)^

Ensuring healthy and safe food access together with food and nutrition security among poorer populations is a critical challenge for Vietnamese policymakers. As is evident in the recent trend towards supermarketization as a remedy for recurrent food safety incidents, food safety is being prioritized at the cost of traditional wet markets for fresh food. These developments make Hanoi an interesting case for studying the impact of retail transformation on the quality of the diets of low-income urbanites.

## III An Interlinked Outcome Approach to Diet and Food Safety

Multiple studies have addressed the relation between dietary intake and disparities in local food environments.^(34)^ Supply-driven approaches are studied in human nutrition and health sciences, and in urban planning and policy.^(35)^ This includes spatial access to food outlets and health-promoting built environments, and how these influence diets.^(36)^ The “food desert” concept was introduced to describe areas with inadequate geographical access to health-promoting foods. The concept has raised structural issues underlying equitable development and the distribution of opportunities supporting health: the retail availability of healthy foods and spatial patterning (spatial/geographical distribution).^(37)^ The food desert concept has expanded to include in-store environmental factors that influence food shopping habits^(38)^ and a temporal dimension in order to understand the association between food access and adverse health outcomes.^(39)^

Along with these supply-driven considerations, the socioeconomic dimensions of accessing food are now also receiving considerable attention. Limited access to healthy foods is associated with socioeconomically disadvantaged populations that are disproportionately affected by NCDs.^(40)^ Economic disadvantage is primarily defined by income as it affects purchasing power, leading to studies that explore price–income barriers to healthy foods. Adding to the concept of the food desert, the concept of “food mirages” has been introduced, referring to regions where grocery stores offering healthy foods are plentiful but prices are beyond the means of poor households, making their food environments functionally equivalent to food deserts.^(41)^ Furthering this analysis, the dual challenge of the urbanization of poverty and of realizing equitable food security drives research aimed at understanding food access as affected not only by income poverty, but also by the cultural and social food practices, habits and routines of consumers.^(42)^ In short, equitable development designed to improve access to healthy foods is receiving attention from multiple disciplines and angles.

However, most studies have been oriented to the global North, while the urbanization of poverty and the challenge of food equity in the context of food retail transitions are most prominent in the global South. Furthermore, most studies define a healthy diet and food environment in terms of nutritional quality and diversity,^(43)^ leaving largely undiscussed the issue of food safety, which is urgent in the emerging economies of the global South.^(44)^ The question should therefore be raised: Can equitable access to safe and healthy foods be achieved in the South using the current retail modernization pathway?

In this study we strive:

- to enhance the body of evidence beyond the global North
- to combine nutritional and food safety aspects in the notion of dietary health (diet diversity and food safety); and
- to link dietary intake with the food retail environment, time–spatial accessibility and socioeconomic affordability outcomes.

**Figure 1:**
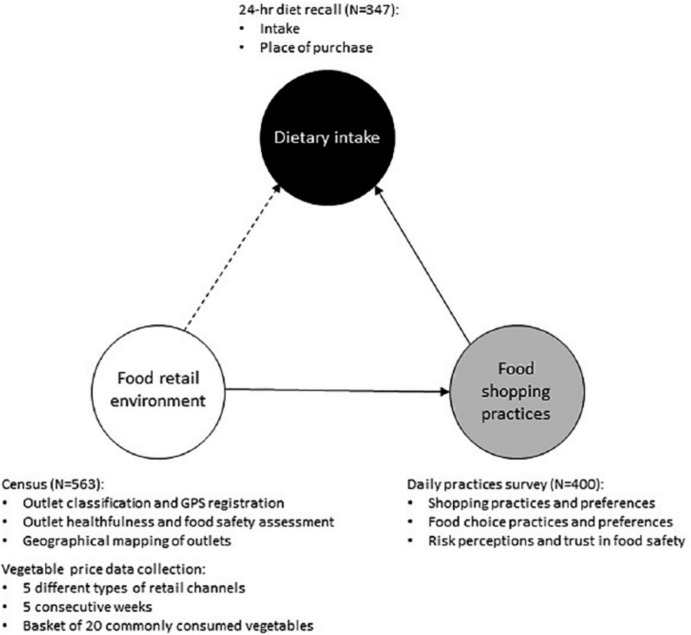
Conceptual framework and methods deployed

In this paper, household food shopping and consumption, and thus dietary intake, are approached as being embedded in the organization of everyday life. We take a novel practice-oriented perspective by including the habitual nature of food consumption^(45)^ and assessing how this is affected in a transforming food retail environment. Building on these social practice approaches to consumption, we explore the relation between the healthfulness^(46)^ of the food retail environment and the dietary intake of low-income urban populations. The hypothesis underlying our research is that the changes in the food retail environment – the aforementioned food retail modernization – impact the diets of low-income urbanites. To address this, we incorporate the logics of everyday life into our conceptual framework (Figure 1), in which outcomes on dietary intake link with the food retail environment through food purchasing practices.

## IV Methods

### a Identifying the target population

With dietary intake as an indirect dependent variable of food retail provisioning, we defined our target population, low-income urbanites, as households with an income of up to US$ 5/person/day. Next, we selected two urban districts in Hanoi with a relatively high percentage of poor households, while excluding the most recently developed suburban areas to avoid a rural or peri-urban bias (see S1 in the online supplementary information for the poverty definition and sampling plan). The study specifically targeted women of reproductive age, since women are primarily responsible for household food purchasing and preparation and are often more nutritionally vulnerable within the household. Being responsible for caring for their families, women often prioritize diet quality for others over themselves, and as a result their diets are likely to be of the lowest quality within a household.

### b Retail outlet census

**Figure 2:**
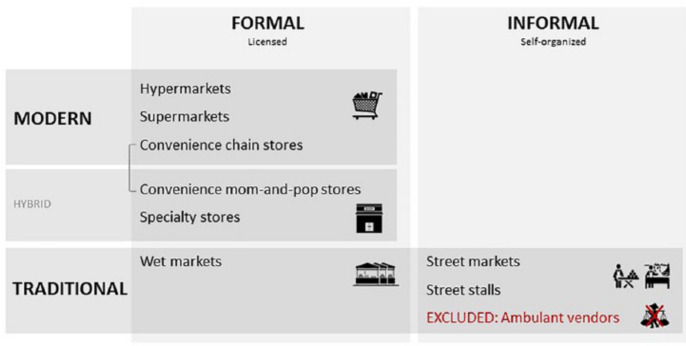
Retail census categorization

To develop a stratification based on food retail environment, we started with a census of food retail outlets, conducted through a street-by-street mapping of commercial food outlets. Each outlet was categorized according to a retail typology (Figure 2), which distinguished between formal (licensed) food retail businesses and informal (self-organized, unlicensed) ones. The typology also differentiated among modern, traditional and hybrid outlets. Within the formal food retail system, we further distinguished four different retail typologies: (i) hyper- and supermarket retail; (ii) convenience retail; (iii) specialty retail focusing on a specific food category such as greengrocers, bakeries and butcher shops; and (iv) wet-market retail. (Hypermarkets, also called superstores, are larger than supermarkets and have a larger section of non-foods, offering a wide range of household goods including electronics.)

Convenience food retail requires some further explanation as Vietnam’s retail modernization policy promulgates the development of chain-based convenience stores. A convenience store carries a limited selection of everyday items, ranging from groceries to drugstore items, like a small version of a supermarket with extended opening hours. Our census distinguishes between modern chain-based convenience stores and more traditional mom-and-pop convenience stores, usually family-owned and typically not franchised. Within the informal food retail system our census also made a distinction between street markets and “street stalls”. The difference is mainly related to whether vendors sell their products collectively (markets) or individually (stalls).

For each outlet, the health dimension of the food assortment available was assessed based on (i) the level of processing of the available foods,^(47)^ (ii) the diversity of food groups offered,^(48)^ and (iii) the diversity of vegetables in particular. This study has a specific focus on vegetables, because vegetables are a relatively low-cost source of nutrition, are a core dimension of the Vietnamese diet and are among the food groups facing the most pressing food safety challenges.^(49)^ Besides, plant-based diets are advocated on a global scale for advancing towards sustainable food futures.^(50)^ With regard to vegetables, for each outlet visual indications that foods on offer were safe for consumption – certification, labelling, branding or marketing communication – were captured at the store, vegetable category and product levels. (See S2 [Food retail census checklist] in the online supplement for further detail.) We refer to these indications as food safety “claims”.

In addition to the census, during five consecutive weeks, the price data of a preselected basket of 20 commonly consumed vegetables were collected within the selected districts. These spanned the five different types of retail channels: supermarket, formal wet market, informal street market, chain-based convenience store, and safe vegetable specialty store (S2).

### c Sampling strategy

**Figure 3:**
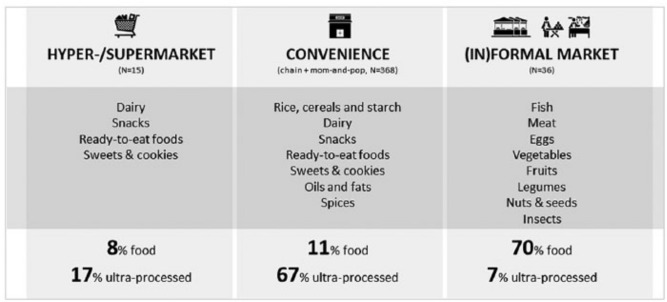
Shopping basket and consumption share across retail outlets

Following the census, we deployed a sample stratification strategy based on the availability of hyper-/supermarkets and formal wet markets within walking distance of 300 metres from respondents’ homes (Table 1). We stratified the sample in this way because these two retail outlet categories are the main focus of the Vietnamese retail modernization policy.^(51)^ All retail outlets identified in the census were geographically mapped using GPS Visualizer (accessible online^(52)^). The results were used to identify geographic areas within the two districts to meet the criteria of the four strata described in Table 1 and illustrated in Figure 3. A door-to-door sampling strategy was deployed for randomized inclusion of households (see S1 in the online supplement for the sampling plan), with a total of 400 households sampled (100 from each stratum). The following inclusion criteria were used during sampling: gender (women only), age (childbearing age, born after 1966), residency (at least two years at the address), household size (excluding single-person households), income (per-capita daily income below US$ 5^(53)^) and role in household food acquisition (primarily responsible).

**Table 1:** Stratification based on food retail outlets within walking distance of the home

Strata	Stratification parameters		Targeted no. of respondents	Actual inclusion	
	Hyper-/supermarket within walking distance (300 metres^(a)^)	Formal wet market within walking distance (300 metres^(a)^)		Household survey^(b)^	Follow-up 24-hour diet recall^(c)^
Stratum 1	Yes	Yes	100	84	71
Stratum 2	Yes	No	100	95	81
Stratum 3	No	Yes	100	92	84
Stratum 4	No	No	100	129	111
Total			400	400	347

Notes

a Since this study focuses on low-income urbanites, the radius of 300 metres from the home on foot was taken as a marker of spatial accessibility. Previous research in Hanoi demonstrated that poorer populations depend on daily food budgeting due to incomes that fluctuate day-to-day and cannot afford to spend money on fuel and parking for shopping purposes. Thus they walk to shops and markets [Wertheim-Heck, Anh et al. (2014)]. We benchmarked this against how the radius is defined in international research: 800 metres for supermarkets and 200 metres for convenience stores, in which the latter refers to walking distance [Raja et al. (2008)]. Previous research included shopping trips by bike [Wertheim-Heck, Anh et al. (2014)]. When bikes are used for shopping this remains within shorter distances of the home, within the “own neighbourhood”. (Important in this respect is the climate: many months are considered too warm for biking.) Hanoi is a city with very limited public transportation options [Hansen (2015)], and public transportation is not used for shopping. Some food retail outlets offer home-delivery services in Hanoi, but the delivery is not free and thus forms a potential affordability barrier for low-income groups.

b Reallocation of households based on retail and household GPS data and the actual distance to the nearest hyper-/supermarket and wet market.

c 87 per cent response rate in follow-up visits.

### d Food shopping practices and preferences survey

A quantitative household survey was conducted that delivered data on household food shopping practices and preferences, food selection processes, vegetable food safety risks and trust perceptions. This included sociodemographic parameters such as age, education, income, household composition and whether the respondent was caring for elderly people and/or children. (See S3 [Survey questionnaire] in the online supplement.) The GPS coordinates of each sampled household were also documented and used to verify the accuracy of stratum allocation (Table 1). The per-capita income was calculated based on the reported household monthly income, divided by the household size and 30 days, then converted from the Vietnamese dong (VND) into US$ using the average historical conversion rate of the month May 2017.^(54)^ The per-capita food budget was calculated based on the reported household food shopping budget and household size and converted from VND to US$ with the same conversion rate.

Following the survey of food shopping practices, a 24-hour diet recall^(55)^ was elicited. During a follow-up visit with the same women, their dietary intake was assessed in terms of dietary diversity^(56)^ – the variety of different food groups consumed during the previous day. All individual food and drink items were recorded and counted in the assessment, including out-of-home consumption^(57)^ and homegrown food items.^(58)^ The method was adapted to also capture processing levels, as well as the sources of food and drinks consumed, to allow for a triangulation of “practice” behaviour with supermarket visits. Two diet diversity indicators were used to measure the quality of the diet. The Diet Diversity Score (DDS) was calculated based on the number of unique food groups out of 10 consumed. We also determined Minimum Diet Diversity (MDD), or the percentage of the population that consumed five or more food groups within a 24-hour period.^(59)^

For the data interpretation we mainly used descriptive statistics, first subjecting the individual datasets to frequency and cross-strata analyses, followed by the combined analysis of datasets and outcome triangulation. (See S4 [Analysis framework] in the online supplementary information.)

## V Main Findings

Below we present the results along the conceptual framework (Figure 1). We start with describing the food retail environment (Subsection Va), which is impacted by food safety-driven policies. We follow in Vb with a description of the dietary outcome based on the respondent sample stratified geospatially, by food outlet proximity. Mediating food environment and dietary intake (Vc), we then proceed in Vd with describing the food shopping practices along time–spatial and quality– affordability dimensions of accessing daily foods, to conclude with the interlinked outcomes of dietary intake and food safety.

### a Food retail environment

The census captured the existing diversity in food retailing (see Table 2 for the overview of food retailers and http://freshstudio.vn/index.php/rd4ddmap for the interactive census map). First, the census confirmed the uneven geographical distribution of the various retail outlets, used to determine the inclusion criteria for stratification. Wet markets are geographically more evenly distributed across the districts, compared to hyper- and supermarkets. Second, the census demonstrated that supermarkets offer a similar assortment of fresh fruits and vegetables to traditional (in)formal markets, but with the difference that the vegetables in supermarkets are offered with visual food safety claims and certificates, which were not observed in the traditional (in)formal markets. However, supermarkets were demonstrated to also offer a wider selection of less healthy ultra-processed foods as defined by Monteiro et al.^(60)^ Third, the census showed that convenience stores are the most widely distributed and dominant retail outlet, accounting for 67 per cent of all food outlets. The census exposes an important distinction regarding the food diversity of more modern chain stores versus traditional mom-and-pop stores: 62 per cent of the chain stores offered fresh vegetables, but only 2 per cent of the mom-and-pop stores. Here it is important to mention that 49 of the 55 chain convenience stores that offered vegetables belong to the same chain retail group, which also includes hyper- and supermarkets. Consequently, 96 per cent of all modern chain stores that offered vegetables supplied them with a food safety claim similar to that in supermarkets.

**Table 2:** Overview of the census of food retail outlets

Food retail category			Count (N=563)	% of outlets	Average % of ultra-processed foods in total food items offered (based on allocated floor and shelf space)	No. of outlets offering fresh vegetables (N=135)	% of outlets per retail category offering fresh vegetables	No. of outlets offering fresh vegetables with visual food safety indication	% of outlets offering fresh vegetables with visual food safety claim^(c)^
Formal	Indoor/storebased	Hyper-/supermarket	16	3%	55%	16	100%	16	100%
		Convenience chain store	89	16%	65%	55^(a)^	62%	53	96%
		Convenience momand-pop store	286	51%	74%	7	2%	3	43%
		Specialty store	104	18%	61%^(b)^	13	13%	9	69%
	Open-air	Wet market	13	2%	25%	13	100%	0	0%
Informal	Open-air	Informal street market	23	4%	25%	23	100%	1	4%
		Informal street stalls	32	6%	78%	15	47%	1	7%

Notes

a 49 of the 55 chain convenience stores belonged to the same retail group (Vinmart).

b Among the stores specializing in fresh produce, ultra-processed foods comprised below 25 per cent of their offer.

c Claims included both more formal certification and more informal claims of clean and safe vegetables, as long as these were explicitly visually communicated at the store, category or individual product level.

### b Sociodemographics

All women included in our sample were of reproductive age (20–50 years old), with 50 per cent aged between 40 and 50. In terms of their final educational qualification, the large majority of respondents (89 per cent) had completed high school (47.3 per cent) or higher (vocational 9 per cent, undergraduate 31.3 per cent, postgraduate 1.5 per cent). The average household size was 4.2 people. 30 per cent of the households had a multigenerational structure that included elderly people (parents or parents-in-law aged 60+); 75 per cent of households had children (36.3 per cent had children aged 0–4 and 54 per cent had children aged 5–12). We only selected households with a per-capita income of US$ 5/ day or less. Table 3 summarizes the sample with respect to household composition, income and food budget. Regarding the stratification of our population under study, there were no significant differences between the variables across the strata.

**Table 3:** Overview of household size, income and food budget

Household size	No. of respondents	Percentage of respondents	Average income (US$)/capita/day^(a)^	Average food budget (US$)/capita/day^(a)^	Percentage of income spent on food
2	15	3.8	4.26	1.80	42.3
3	78	19.5	4.30	1.82	42.3
4	182	45.5	3.94	1,63	41.4
5	79	19.8	3.53	1.39	39.4
6	38	9.5	3.11	1.30	41.8
7^(c)^	8	2.0	2.70	1.10	40.7
Total	400	100	3.64^(b)^	1.59^(b)^	41.2^(b)^

Notes

a Currency conversion on 15 May 2017: VND 22,689 = US$ 1.

b Relative weight (average income was weighted by the number of respondents).

c Largest household size included in this study.

### c Dietary intake

There was an 87 per cent follow-up response rate to the dietary intake survey (Table 1), with households not continuing mostly due to ill health or relocation. MDD was reached by 75 per cent of women, with an average DDS of 5.3 food groups consumed in the past 24 hours (only 0.3 of a food group above the inclusion cut-off of five food groups). Although it was expected that diet diversity would be influenced by geographical proximity to different formal retail outlets, our analysis showed no significant difference in diet diversity across the different strata (Table 4).

**Table 4:** Dietary quality across strata

Diet quality indicators	Stratum 1 (n=71)		Stratum 2 (n=80)		Stratum 3 (n=84)		Stratum 4 (n=112)		Overall (n=347)		P value	Test
	Mean	SD	Mean	SD	Mean	SD	Mean	SD	Mean	SD		
Number of food groups (out of 10) consumed by women	5.0	1.2	5.1	1.3	5.4	1.3	5.5	1.2	5.3	1.2	0.06	ANOVA
% of women of reproductive age who consumed .5 food groups	67.6		72.5		77.4		79.5		74.9		0.29	χ^2^
Proportion of daily energy intake from ultra-processed foods	4.9	10	5.4	10.8	6.4	10.9	7.2	11.3	6.1	10.8	0.21	Kruskal-Wallis

NOTE: SD = standard deviation.

Nearly all the women (96 per cent) had consumed meat, poultry or fish and nearly 80 per cent had consumed dark green leafy vegetables in the previous 24 hours. Fruit consumption was quite high (84 per cent); however, less than 2 per cent of the women had consumed a vitamin A-rich fruit. Women who reached MDD were more likely to have consumed legumes (seven times), nuts and seeds (five times), dairy (four times), fruits (1.7 times) and vitamin A-rich foods (1.3 times) than those below MDD.

Our results reveal that only 8 per cent of the foods consumed were sourced from supermarkets. Traditional (in)formal markets were the most important source of food (48 per cent), followed by convenience stores (23 per cent), specialty single-item stores (15 per cent) and supermarkets (8 per cent). Only 2 per cent of foods consumed were homegrown and 4 per cent were obtained from alternative sources outside of the Hanoi food retail environment, including via contacts who enabled foods to be sourced directly from the rural production areas. There was no significant difference between strata regarding where women sourced foods except for own production, where Stratum 4 (who had no wet market close by) sourced more (3 per cent) and Stratum 1 (who lived close to wet markets) sourced less (1 per cent). Most of the ultra-processed foods consumed were purchased from convenience stores (67 per cent), predominantly mom-and-pop stores, followed by supermarkets (17 per cent), traditional wet and street markets (7 per cent) and specialty stores (6 per cent). There was no significant difference in ultra-processed food consumption between strata.

### d Food shopping practices

We correlated dietary intake with the food retail environment mediated through food shopping practices along two dimensions: (i) time–spatial and (ii) quality–affordability.

#### Time–spatial dimension of accessing foods and dietary intake

The comparable diet diversity observed across strata is explained by the similar food shopping patterns, regardless of proximity to supermarkets or wet markets. The dominant food shopping practice entails shopping daily (92 per cent) in the morning (55 per cent before 8 am / 90 per cent before 11 am) at traditional (in)formal markets (88 per cent), mostly accessed on foot (67 per cent) or by motorbike (27 per cent). Whether respondents reported shopping at formal wet markets or informal street markets was clearly geographically defined. For the strata that had a wet market within 300 metres of the home (Strata 1 and 3), 60 per cent purchased most of their foods at the formal wet market and 38 per cent at a nearby informal street market. In comparison, in Strata 2 and 4, which had no wet market close by, 80 per cent of foods were purchased from an informal street market, and 18 per cent at a formal wet market. Retail outlet proximity thus influences food shopping practices, but this does not include supermarkets, which were not widely utilized by the sample (Table 5). Only 7 per cent of the respondents were classified as regular supermarket shoppers (at least once a week, N=28). Of this small group, 75 per cent did not have a supermarket within walking distance.

**Table 5:** Practices and preferences related to shopping outlets, by stratum (%)

Walking distance to outlets		Stratum 1	Stratum 2	Stratum 3	Stratum 4
		SM+WM	SM	WM	Neither
**Practice:** Where do you buy most foods for your household?	Hyper-/supermarket (SM)	0.0	0.0	0.0	0.8
	**Formal wet market (WM)**	**57.1**	22.1	**63.0**	14.7
	**Informal street market**	39.3	**76.8**	35.9	**82.9**
	Convenience store	2.4	1.1	0.0	1.6
	Specialty store	1.2	0.0	1.1	0.0
	Street vendor	0.0	0.0	0.0	0.0
**Preference:** Where would you prefer to buy food for your household?	Hyper-/supermarket (SM)	4.8	0.0	7.6	3.9
	**Formal wet market (WM)**	**53.6**	23.2	**60.9**	18.6
	**Informal street market**	38.1	**75.8**	23.9	**75.2**
	Convenience store	2.4	1.1	0.0	0.5
	Specialty store	1.2	0.0	1.1	0.0
	Street vendor	0.0	0.0	0.0	0.0

The results indicate that low-income consumers in Hanoi do not frequent supermarkets for their main food shopping, even when these are located close to their home or when they are not close to a traditional retail outlet. Of all 179 households with a supermarket within walking distance (Strata 1 and 2), none reported supermarkets as their main source of food. Overall, 98 per cent of respondents reported shopping primarily at (in)formal markets, which account for only 6 per cent of total food retail outlets within the local food environment and 23 per cent of total food retail outlets offering fresh vegetables in the census.

We also assessed the preferences in food shopping. The preferences corresponded – with only a slight difference – to the aforementioned practice of shopping every day (preferred by 94 per cent) in the morning (preferred by 93 per cent) at traditional (in)formal markets (preferred by 93 per cent: 37 per cent formal wet markets and 56 per cent informal street markets).

The primary reasons for shopping at traditional (in)formal markets were habit (100 per cent), the wide assortment offered (99 per cent), the convenient close-to-home location (98 per cent) and the enjoyment of shopping at the market (97 per cent). Further reasons included the availability of a wide variety of fresh nutritious foods (86 per cent), the low selling price (80 per cent), and trusted food safety (77 per cent), in which food safety is ensured by choosing seasonal products that look fresh, followed by knowing where the vegetables were produced. Personal contact with the vendor (66 per cent) and a convenient location “on the way” from home to work/school (60 per cent) appeared less important. These percentages refer to the total study population as we did not measure significant differences across the strata. Only 4 per cent (N=16) of the respondents reported preferring to shop in hyper-/supermarkets, compared to 56 per cent at informal street markets (Table 5).

#### Quality–affordability dimension of accessing foods and diet diversity

The mean household income was calculated at US$ 3.64/person/day (median US$ 3.67/person/day).^(61)^ The range was between US$ 1.38 and 4.90/person/day, which corresponds with the lowest two income quintiles of Hanoi.^(62)^ The mean per-capita daily food budget was calculated at US$ 1.59 (median US$ 1.65), which indicates that on average 41.2 per cent of the household income was spent on food for in-home consumption (Table 3).

To assess the quality–affordability dimension of accessing food and dietary intake, we zoomed in on the category of vegetables. Although vegetables are not the most expensive ingredient of a daily meal, they are nevertheless reported to make up a substantial part of the total food expenditure.^(63)^ Significant differences in measured price levels of vegetables were observed between different retail outlets, particularly between (in)formal markets and specialty safe vegetable stores. Supermarkets are on average 35 per cent more expensive than traditional markets (wet markets or street markets). Specialty stores with explicit food safety claims are about 50 per cent more expensive than supermarkets and charge nearly double the price of traditional channels.

The household survey uncovered a preference across strata that food should be affordable and provide good value for money, but for the large majority, “cheap” was not an important selection criterion. However, at the same time, nearly 86 per cent of the respondents considered the prices of safe food too high. When further exploring the affordability dimension, we combined reported income and food budget with cross-channel price indexation. We calculated the effects of vegetable price differences across channels as a percentage of the food budget.^(64)^ This indicated that when vegetables were purchased at traditional (in)formal markets they accounted for about 19 per cent of the food budget, compared to supermarkets (27 per cent) and specialty (safe vegetable) stores (37 per cent). With only three respondents (less than 1 per cent) shopping at specialty stores for safe vegetables, we may conclude that, besides being not widely distributed in the areas under study, these are not affordable for low-income populations.

The main price/quality differentiator across retail channels was food safety. The census uncovered that formal food safety guarantees were most commonly found in modern retail channels, whereas (in)formal markets did not provide for food safety certification, labelling or branding (Table 2). Nearly all vegetables (96.3 per cent) sold through the modern trade channels of hypermarkets, supermarkets and chain convenience stores were offered with food safety claims. In the traditional trade through formal wet markets, informal street markets and street stalls, visual food safety claims were largely absent (less than 3.8 per cent).

Although 98 per cent of the respondents were concerned about the safety of vegetables, and 61 per cent believed that vegetables offered in supermarkets could generally be considered safer than the vegetables offered in wet markets, vegetables were predominantly purchased at traditional (in)formal markets. Buying at supermarkets was considered “rather safe” (67.5 per cent), but was also considered expensive (85.1 per cent). On the other hand, 62 per cent of respondents disagreed with the statement that shopping at wet markets was expensive, and reported being confident in their ability to avoid food safety risks as they trusted their own risk-mitigation strategies/capabilities, including vendor selection, product selection and food cleaning practices.

Along with safety, “freshness” was regarded as an important food quality indicator. Vegetables sold through modern channels were considered less “fresh” than vegetables purchased at (in)formal markets.

The level of food processing was also considered an indicator of food quality. Our research uncovered that modern retail outlets not only offered a higher percentage of ultra-processed foods than traditional (in) formal markets (>60 compared to <25 per cent), but that these channels were mainly frequented for purchasing such foods, with 92 per cent of the respondents who shopped less than once a week at supermarkets primarily visiting these outlets to purchase processed foods, like snacks and sweets. The 24-hour dietary recall further uncovered that although only a limited amount of food (19 per cent) was purchased in supermarkets and convenience stores, these outlets contributed to 84 per cent of the ultra-processed foods consumed. In contrast, traditional (in)formal market channels, which were frequented on a daily basis, contributed only 7 per cent of the less healthy ultra-processed food category (Figure 3).

## VI Discussion

The survey uncovered that 94 per cent of the foods consumed were obtained through retail channels, which justified our focus on food shopping practices. Our results demonstrate that supermarkets do not (yet) contribute to more healthy diets for low-income urbanites. When formal wet markets are absent, informal vending structures, most importantly informal street markets, are preferred over supermarkets. Our 24-hour diet recall confirmed this preference for traditional (in)formal markets, as Stratum 1, which had both wet markets and supermarkets within walking distance, sourced 6 per cent more foods from informal street markets than the average of the other three strata. These shopping practices resulted in diets that still strongly reflected Vietnamese food culture, containing relatively high quantities of fresh fruits and vegetables when compared to global and regional averages, but with increasing quantities of animal-sourced foods.^(65)^

Our study uncovers how the impact of food retail modernization on the diets of low-income urbanites is mediated through the everyday shopping practices in which time–spatial and quality–affordability dimensions exclude modern store-based alternatives. This is explained by the convenience of open-air (in)formal markets in the routine of early-morning, everyday fresh food shopping close to the home. The importance of considering food affordability, and the subsequent impact on household expenditure of changing access to certain retail outlets, was indicated by the significant differences in vegetable prices measured across retail outlets. The affordability dimension requires further research as the current measurements included only a limited number of outlets.

This paper demonstrates the complexity of development effects. Policymakers are confronted with the social justice dilemma of improving food safety without jeopardizing a traditionally healthy and diverse diet. Based on our results, we suggest that the dual public responsibility for ensuring continued access to nutritious and safe foods requires a more diverse retail approach than that planned under the current supermarketization policies. An obvious benefit of the promotion of modern retail chains is their strong and demonstrated interest in promulgating safe foods. However, our results also demonstrate how retail modernization currently plays out negatively for low-income urbanites, highlighting the current incompatibility of aiming for improved food safety and diet quality through these modern channels, potentially at the cost of traditional outlets.

In preventing the creation of nutrition deserts or mirages for low-income consumers, food safety policies should recognize the importance of allowing for a versatile and hybridized food retail environment to evolve. On the one hand, policy should focus on how to mitigate the undesirable dietary effects of supermarketization, which lead to the increased prevalence of NCDs. Although low-income consumers in Hanoi are not yet widely utilizing modern retail outlets, in their well-established shopping practices the relatively high contribution of supermarket and convenience outlets to the consumption of ultra-processed foods hints at a potential health risk. The proliferation of transnational retail corporations is strongly associated with increased availability of ultra-processed foods.^(66)^ This has been demonstrated to negatively impact the uptake of nutritious fresh foods in the diet, which leads to increases in overweight, obesity, cardiovascular disease and other NCDs.^(67)^

Instead of depriving low-income consumers of traditional fresh food vending infrastructure, policy should look for ways to renovate and upgrade existing facilities and (in)formal organizational structures with deficient food safety standards. A study in Thailand uncovered that frequent market shopping was associated with increased vegetable intake, whereas frequent shopping at supermarkets and convenience stores was associated with the consumption of six “problem foods”: soft drinks, snack foods, processed meats, Western-style bakery items, instant foods and deep-fried foods.^(68)^ Also, studies in Vietnam have highlighted the role of informal vending structures beyond economic transactions.^(69)^ Thus, policy should allow for transformations of retail in its different and diverse forms, including traditional and informal ones.

It is important to highlight that the effects of retail modernization policies on dietary diversity are contextual. Supermarket-based food provision is demonstrated to play out differently for low-income populations in different contexts, with disparate associations of supermarket shopping with obesity between Western and developing-country contexts.^(70)^ In seeking to advance and enhance food retail safety, hygiene and nutritional quality in a socially just way, we argue that modernization theories and food retail policies should be contextually evidence-based; broad assumptions such as “supermarkets provide better food environments” should be avoided without local empirical evidence.

Our research provides insights into how increasing retail modernization does not yet influence diet diversity and shopping practices in the specific context of low-income consumers in Hanoi, Vietnam. In reconciling the apparently competing priorities of food safety and nutrition for low-income consumers, there is a need for intervention studies that consider dynamic societal interactions. These should allow for co-creation that actively involves consumers, producers, retailers and policymakers within the local food business ecosystem, to develop tailored retail intervention strategies that consider the multiple foodways – cultural, social and economic food practices and preferences – of low-income populations. Currently, there is little to no evidence available on actual levels of food safety or toxicity that would allow for a comparison between different retail outlet typologies. Biochemical analysis of foods from different retail outlets would allow for a more nuanced understanding of how the urban poor’s perception of food safety in modern vs. traditional retail outlets measures up with reality. These data would be useful to better inform both policymakers and consumers.

This paper reflects the first results from an ongoing research project exploring drivers of food choice of low-income consumers in Hanoi, based on a stratification defined by home locations and retail proximity. However, neighbourhood definitions of the food environment are crude measures that might be misaligned with broader lifestyle patterns. Although we included transportation modes, shopping frequency, time of shopping and other convenience indicators, this patterned approach does not yet capture the heterogeneity in wider daily activities, let alone future lifestyle changes that are expected with continued urbanization and modernization of Asian cities. It was difficult to discern drivers of access and utilization of supermarkets because only a small number of households actually accessed these modern retail outlets. However, we did find that supermarket distance from the home appeared not to be the most relevant indicator of accessibility. Ongoing research seeks to further deepen our understanding of access to safe and healthy foods by capturing lifestyle dynamics, including intra-household dynamics and generational shifts over time, in order to inform socially just policies.

## VII Conclusions

Food retail modernization is not (yet) utilized in the well-established shopping practices of low-income consumers in Hanoi. Our results demonstrate that from both time–spatial and quality–affordability perspectives, supermarkets do not contribute to more healthy diets for low-income urbanites. Our initial hypothesis about the impact of the food retail modernization on the dietary intake, in terms of dietary diversity, proved negative. Distance to supermarkets did not change shopping practices. Although the census provided insights on the uneven distribution of supermarkets across all strata, the foods consumed mainly originated from traditional outlets. In the absence of formal wet markets, dietary diversity was maintained through close-to-home informal vending structures. As such, it was not surprising to find that households’ distance to either supermarkets or wet markets did not influence diet diversity – no significant difference was found between strata in either DDS or MDD. Supermarket shopping is not only considered inconvenient and time consuming, but the safe foods offered are also considered rather expensive and less fresh. Supermarkets currently contribute mainly to the consumption of ultra-processed foods.

This study unpacks the competing priorities of nutrition and food safety in governing food security for low-income urbanites. On the one hand, the results confirm the effective direction of the dominant food safety-oriented retail modernization policies. Our research demonstrates that food safety guarantees are indeed omnipresent in modern retail channels; all hyper- and supermarkets and nearly all convenience chain stores offer vegetables with visual food safety guarantees. Moreover, the results show that these outlets offer a similar assortment of fresh vegetables to traditional outlets.

However, the findings also demonstrate that low-income urbanites rarely frequent these modern retail channels, even when a supermarket is very close to their home. Traditional food retail structures, in the form of (in)formal markets, appear crucial in maintaining a minimally adequate diverse diet, although they do not provide general or formalized food safety guarantees. When formal wet markets are absent, supermarket proximity is shown to have no impact on diet diversity because informal street markets act as fill-ins for similar foods.

The women in our study were from low-income households and were prevented in practical terms from purchasing fresh foods with formal food safety guarantees. Supermarkets presented multiple access barriers (along combined time–spatial and quality–affordability dimensions) and thus barely contributed to their daily diet. Given this reality, the presence (or absence) of supermarkets and their food assortment do not yet seem to have direct effects on diet quality of low-income consumers in Hanoi. However, this may change in the future when traditional market vending is further reduced, and especially when informal street vending is more forcefully repressed by supermarketization policy.

The struggle of low-income urbanites with food safety is a well-recognized problem in Vietnam and affects people throughout Asia. Although it is not contested that traditional markets are often less hygienic than supermarkets and lack adequate control mechanisms, this research demonstrates the limits to pushing modernization by banning traditional vending structures as a remedy for recurrent food safety incidents. Our research demonstrates that these one-dimensional ideal-type policies have limited success in improving access to certified-safe foods among low-income residents, or for improving diet diversity. They fail to produce socially inclusive food retail infrastructure. Moreover, our study provides a hint at how these policies, when successful, might jeopardize dietary quality for low-income urbanites through two pathways: depriving them of access to nutritious foods and stimulating less healthy diets by increasing consumption of ultra-processed foods. Thus, transforming the food environment without considering the food shopping practices, especially of low-income citizens, might result in unwanted outcomes in terms of dietary intake.

## Footnotes

1 Quote from a focus group participant in a project on supplying lower-income groups with vegetables certified as safe. See Wertheim-Heck et al. (2014).

2 FAO (1996).

3 Tefft et al. (2017).

4 NCD Risk Factor Collaboration (NCD-RisC) (2017); Boonchoo et al. (2017); Do et al. (2017).

5 Mathur (2014); Ravallion et al. (2007); Kanbur and Zhuang (2013).

6 Mohiddin et al. (2012).

7 Ortega and Tschirley (2017); World Bank (2017).

8 Reardon and Timmer (2012); Reardon and Timmer (2014).

9 Reardon (2006); Wrigley and Low (2010); Fuchs et al. (2011).

10 Moore (2013); Cohen (2018).

11 Maryama and Trung (2007); Wertheim-Heck et al. (2015).

12 Friel and Baker (2009); Mohiddin et al. (2012); Ruel (2001).

13 Pingali (2007); Herforth and Ahmed (2015).

14 Wertheim-Heck, Vellema et al. (2014).

15 *VietNamNet Bridge* (2017).

16 GSO (2009–2016).

17 Wertheim-Heck, Anh et al. (2014).

18 Wertheim-Heck et al. (2015).

19 Khan and Hoan (2008); Lachat et al. (2009).

20 MoPI (2011); MoIT (2012).

21 Moustier (2006); MoIT (2009); Dries et al. (2013).

22 Maruyama and Trung (2007); Moustier (2006); Dries et al. (2013); Wertheim-Heck et al. (2015).

23 MoIT (2009).

24 Wertheim-Heck et al. (2015).

25 Havelaar et al. (2015); World Bank (2017).

26 Reardon (2006); Wrigley and Low (2010).

27 Wertheim-Heck, Vellema et al. (2014).

28 Van Hoi et al. (2009); World Bank (2017); Ha et al. (2019).

29 Shepherd and Tam (2008).

30 Wertheim-Heck, Vellema et al. (2014); Ha et al. (2019).

31 Figuié et al. (2004); Mergenthaler et al. (2009); Mergenthaler et al. (2006); Ha et al. (2019).

32 Dries et al. (2013).

33 Ortega and Tschirley (2017).

34 Southerton et al. (2004); Hereforth and Ahmed (2015); Leite et al. (2018).

35 Murphy et al. (2017).

36 Moore et al. (2008); Black, Moon et al. (2014); Morland (2014).

37 Páez et al. (2010); Lamichhane et al. (2013).

38 Black, Ntani et al. (2014).

39 Chen and Clark (2013); Widener and Shannon (2014).

40 Wang et al. (2007).

41 Breyer and Voss-Andreae (2013); Sullivan (2013).

42 Alkon et al. (2013); Samanta (2015).

43 Herforth and Ahmed (2015); Friel and Baker (2009).

44 Ortega and Tschirley (2017).

45 Warde and Southerton (2012).

46 Páez et al. (2010); Lamichhane et al. (2013).

47 Monteiro et al. (2016).

48 FAO and FHI 360 (2016).

49 Ha et al. (2019).

50 Willett et al. (2019).

51 MoIT (2009).

52 Uyen and Wertheim-Heck (2018).

53 Regarding sociodemographic triangulation for income underreporting, income underreporting is a prevalent complication in poverty research and this does not exclude this research. Although we based the participant inclusion on a per-capita income of ≤US$ 5/day, the actual income level may be higher. To assess whether we included more vulnerable populations, we benchmarked the average household size of 4.2 persons and 30 per cent extended households in our research against the generic urban population statistics of Vietnam, which report substantially lower household sizes: average household size of 3.7 persons (GSO, 2009) and 14.9 per cent extended households [Guilmoto and de Loenzien (2015)]. This indicated that our household profile indeed corresponds with the profile of lower income classes.

54 http://www.oanda.com.

55 Gibson and Ferguson (2008).

56 FAO and FHI 360 (2016).

57 Tuyen et al. (2004).

58 Wertheim-Heck and Spaargaren (2015).

59 FAO and FHI 360 (2016).

60 Monteiro et al. (2016).

61 This corresponds with Hanoi Income Quintile 2: Hanoi average per-capita income at current prices by income quintile (2016 preliminary GSO statistics, http://www.gso.gov.vn).

62 GSO (2016).

63 This excludes expenditure on meals outside of the home, which accounted for 4.5 per cent of total food expenditure among poor groups in Hanoi. This would mean that the average total food budget amounted to 46 per cent of the average income. Centre for Global Food and Resources (undated).

64 The portion (%) of the daily food budget of US$ 1.5/ person we calculated is based on price data collection and an average daily vegetable shopping amount of 377 grams (290 grams/person intake plus 30 per cent waste = 377 grams purchased/person). This is based on previous daily shopping journal research: Wertheim-Heck, Vellema et al. (2014). The calculation was based on a limited selection of vegetables that was available across all channels during the price data collection.

65 Development Initiatives (2018).

66 Hawkes (2005); Hawkes (2006).

67 Moubarac et al. (2012); Canella et al. (2014); Popkin (2014); Rauber et al. (2015); Luiten et al. (2016); Taillie et al. (2016); Hyseni et al. (2017); Poti et al.

68 Banwell et al. (2012); Kelly et al. (2014).

69 Nguyen et al. (2013); Kawarazuka et al. (2017).

70 Larson et al. (2009); Demmler et al. (2017).
