# Supplementary material for: Food safety and nutrition for low-income urbanites: exploring a social justice dilemma in consumption policy
Source: Reg Environ Change. 2019 Oct;31(2):397–420. doi: 10.1177/0956247819858019 (PMC7340485; doi:10.1177/0956247819858019)
Supplement: Supplementary file 1 [file EU-2019-0956247819858019-s1.pdf]

Food safety and nutrition for low-income urbanites: exploring a social justice dilemma in consumption policy

Supplementary information

S1: SAMPLING PLAN

The following sampling strategy was the basis for (i) the household survey, (ii) the nutrition survey and 24-hour diet recall, and (iii) the qualitative research. The strategy was divided into five steps, of which steps 1 to 3 were completed before the start of the survey fieldwork, while steps 4 and 5 were addressed during the field research.

STEP 1: POVERTY DEFINITION

In this research a cut-off point for poverty of US$ 5/person/day was used. This built on the Vietnam Household Income Band (HIB),[(1)] which follows the income classifications used by the Vietnam General Statistics Office.[(2)] These classifications are well understood by the local population and were used in previous research.[(3)] For discussing the bottom of the pyramid, internationally different cut-off points are used (from US$ 1.25/person/day [World Bank] to up to US$ 8/person/day.[(4)] The World Bank definition of poverty at US$ 1.25/person/day appears too narrow for an Asian urban context, given the price levels of basic necessities and high inflation rates in Hanoi, as well as the fact that the urban context does not allow for food self-provisioning (like in rural areas).

Aiming to understand the potential impact of food retail on dietary intake through shopping practices, this study included a population with minimum purchasing power, but below the minimum wage. The minimum wage in 2017 in Hanoi was US$ 5.75/person/day.

Respondent inclusion was based on monthly household income and household size in order to allow inclusion based on per-capita income levels. See the respondent inclusion question in Table 1, which was used in the household survey below:

Table 1

**Q8**	**A**	**How many persons are living within your household?** **[Single answer]**								
			2 pers	3 pers	4 pers	5 pers	6 pers	7 pers	8 pers	9+ pers
**B**		Code	1	2	3	4	5	6	7	8
**What is your household's monthly income?**	Less than VND 3 mln	1	Continue							
	3,000,000–4,499,999	2								
	4,500,000–5,499,999	3								
	5,500,000–6,499,999	4								
	6,500,000–7,499,999	5	STOP	Continue						
	7,500,000–8,499,999	6	STOP							
	8,500,000–9,499,999	7	STOP							
	9,500,000–10,499,999	8	STOP							
	10,500,000–11,499,999	9	STOP	STOP	Continue					
	11,500,000–12,499,999	10	STOP	STOP						
	12,500,000–13,499,999	11	STOP	STOP						
	13,500,000–14,999,999	12	STOP	STOP	STOP	Continue				
	15,000,000 and over	13	STOP							

STEP 2: DISTRICT SELECTION

Districts were purposively selected based on the following criteria:

1. Exclusion of recently developed urban areas on the outskirts of urban Hanoi (like Ha Dong)
2. Focus on the more inner urban districts
3. Exclusion of atypical districts: Tay Ho (expatriate community) and Hoan Kiem (tourist area)
4. Final district selection based on:
   - Retail density (supermarket)
   - Information readily available on markets (HealthBridge Livable Cities Project – Preserving Public Markets: https://healthbridge.ca/projects/entry/livable-cities-vietnam)
   - Size in square kilometres (feasibility for census)
   - Percentage of the population that was poor according to GSO statistics (although we did not focus on the official poor living below US$ 1/person/day)

Out of the five relevant urban districts (Table 2), two districts were selected: Dong Da and Ba Dinh.

- Dong Da has the highest population density and the highest number of supermarkets.
- Ba Dinh is a very central district (Old Hanoi), with the highest proportion of poor households.

These districts and others are mapped in Figure 1.

Table 2

District	No. of wards	Size (km^2)	Population	Population density (no. of people per km^2)	Proportion in extreme poverty (living on less than US$ 1/person/day	No. of supermarkets
**Ba Dinh**	**14**	**9.224**	**242,800**	**26,323**	**0.92%**	**10**
Cau Giay	8	12.04	251,800	20,914	0.12%	10
**Dong Da**	**21**	**9.96**	**408,000**	**40,964**	**0.55%**	**15**
Hai Ba Trung	20	14.6	378000	25,890	0.66%	9
Thanh Xuan	11	9.11	259000	28,430	0.25%	5

NOTE: Selected districts in bold print.

SOURCES: GSO (2012), *Household Living Standard Survey*, General Statistics Office Of Vietnam; Hanoi Statistics Office (2013), *Statistics of Hanoi Population & Labour*.

Figure 1

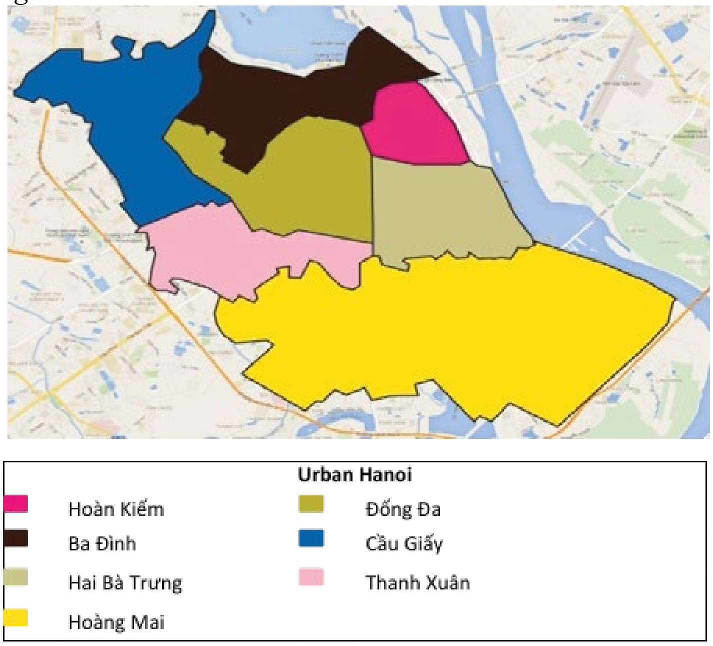
Urban Hanoi
Hoàn Kiếm
Đống Đa
Ba Đình
Cầu Giấy
Hai Bà Trưng
Thanh Xuân
Hoàng Mai

STEP 3: SELECTION OF SAMPLING AREAS

In the districts Dong Da and Ba Dinh, a census of the retail outlets was conducted. The census data provided the basis for the respondent inclusion. Based on the GIS census mapping, concentric circles were drawn around the identified hyper- and supermarkets and formal wet markets to identify areas that fit the four sampling strata, shown in Figure 2 and Table 3 below.

Table 3

Respondents	Supermarket within walking distance (300 metres)	Formal wet market within walking distance (300 metres)
Group 1 (N=100)	Yes	Yes
Group 2 (N=100)	Yes	No
Group 3 (N=100)	No	Yes
Group 4 (N=100)	No	No

Figure 2

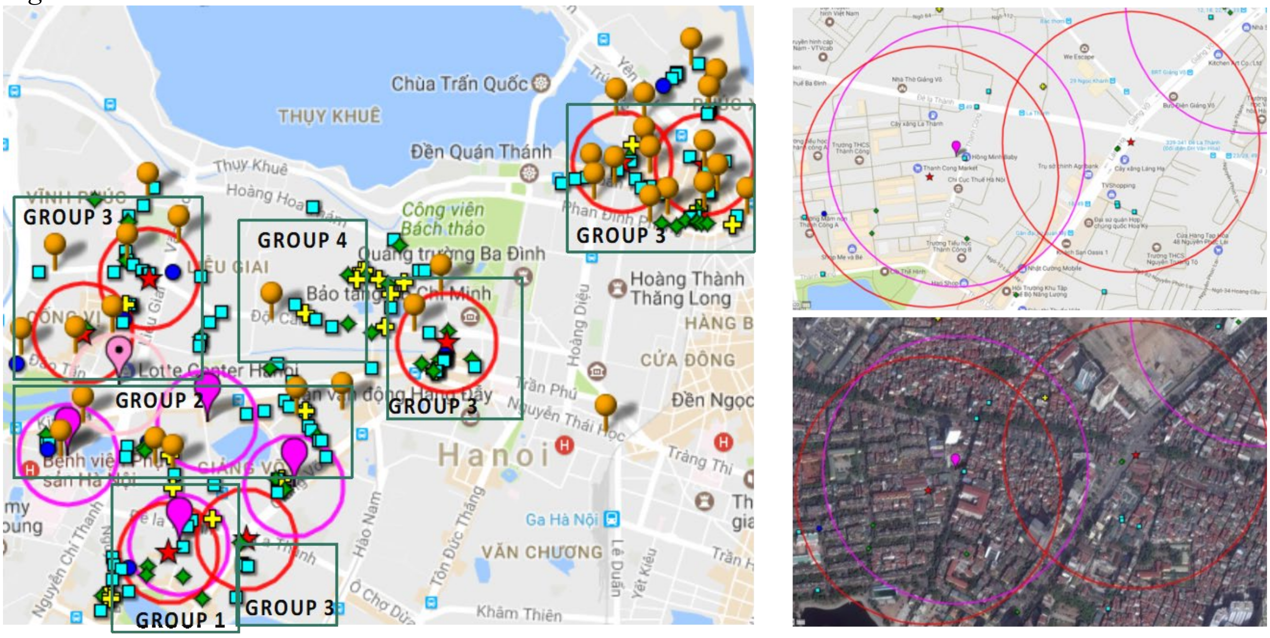

The enumerators were assigned to a specific area. The enumerators were divided over the two districts, but the district was not a variable in our analysis. A balanced distribution between the two districts was preferred, but because the district was not a variable in our data analysis, it was not a serious problem that for practical reasons during the excution, slightly more or fewer households were included per group in one district compared to the other, as long as the total numbers per stratum were met. Table 4 shows the numbers of respondents and presence of markets for the two selected districts.

Table 4

	Respondents	Supermarket within walking distance (300 metres)	Formal wetmarket within walking distance (300 metres)
**Ba Dinh**	Group 1 (N=50)	Yes	Yes
	Group 2 (N=50)	Yes	No
	Group 3 (N=50)	No	Yes
	Group 4 (N=50)	No	No
**Dong Da**	Group 1 (N=50)	Yes	Yes
	Group 2 (N=50)	Yes	No
	Group 3 (N=50)	No	Yes
	Group 4 (N=50)	No	No

STEP 4: HOUSEHOLD/RESPONDENT INCLUSION

The survey was a door-to-door survey in which participants were randomly selected within the aformentioned areas. The enumerator assigned to a specific area randomly selected a street to start the fieldwork, went to a random house or apartment building on the street, and approached respondents with the following strategy:

- No more than three households/apartments were included within the same apartment building.
- Only every third apartment within an apartment building was included.
- Only every third building on the street was included.
- Further participant inclusion criteria were included in the survey questionnaire.

Proceedings in short:

1. Completed survey: continue to the third next apartment within the building (max. of three apartments per building), or to the third next building.
2. Unqualified survey: continue to the next apartment within the building or to the next building.

STEP 5: TIME SLOTS

To avoid a bias based on timing of visits during the day, the enumerators operated in three different timeslots (Table 5).

Table 5

Period	Timeslot*	Explanation
Morning	7–11 am	Previous research has indicated that most people are shopping in the early morning. This timeslot thus risks excluding morning shoppers.
Afternoon	2–6 pm	The afternoon allows for the inclusion of people without a job, who are mostly shopping in the morning and using the cooler evening hours for other activities.
Evening	6–10 pm	PREFERENCE:<br>Most people are expected to be at home, and it is further expected that women responsible for household shopping and cooking will have more time available in the evening than during daytime.

**Lunch time was excluded*

[1] Nielsen (2013), *Know Your Consumers Grow Your Business: 2013 Pocket Reference Book Vietnam*; also Nielsen (2014), *Know Act Grow: Driving Smarter Business Decisions in Vietnam*, accessed 10 May 2019 at http://www.nielsen.com/content/dam/nielsenglobal/vn/docs/Reports/2014/Know-Act-Grow-nov-2014.pdf.

[2] GSO (2010), *Results of the Vietnam Household Living Standard Survey 2010*, General Statistics Office Vietnam, accessed 10 May 2019 at https://www.gso.gov.vn/default_en.aspx?tabid=515&idmid=5&ItemID=12426.

[3] Wertheim-Heck, S C O, H T L Anh, H T T My, M Klaver and P T T Huong (2014), *Reaching Lower Income Groups with Safe and Healthy Foods - Mission Possible? Insights into the Consumption of Lower Income Consumers in Urban Hanoi*, Fresh Studio, Hanoi, accessed 1 February 2019 at http://www.freshstudio.vn/images/media-archive/Publications/2014/20140612%20Reaching%20lower%20income%20groups%20with%20safe%20and%20healthy%20food%20-%20mission%20possible.pdf.

[4] BoP Innovation Center (2013), *BoP Innovation Cycle: The Process of Innovation to Create Inclusive Business.*
