# Supplementary material for: Food safety and nutrition for low-income urbanites: exploring a social justice dilemma in consumption policy
Source: Reg Environ Change. 2019 Oct;31(2):397–420. doi: 10.1177/0956247819858019 (PMC7340485; doi:10.1177/0956247819858019)
Supplement: Supplementary file 2 [file EU-2019-0956247819858019-s2.pdf]

Food safety and nutrition for low-income urbanites:
exploring a social justice dilemma in consumption policy

Supplementary information

S2: FOOD RETAIL CENSUS CHECKLIST

Census no:	
Date:	Time:
Enumerator statement<br><br>I have finished the census according to the instructions of the project<br><br>(Signature and full name)	QC statement<br><br>I have checked this census according to the instructions of the project<br><br>(Signature and full name)

**1**	District:	☐ Ba Dinh ☐ Dong Da			
**2**	Name of shop:				
**3**	Address:	GPS coordinates	No	Alley	Street/ road
**4**	Contact details:	Telephone:		Email address:	
**5**	Type of shop:	☐ Hypermarket ☐ Supermarket ☐ Convenience store<br>☐ Mom-and-pop store ☐ Specialty food shop<br>☐ Wet market ☐ Street market ☐ street food stall			

**6**	Shop operational since:	☐ < 1 year ☐ 1-2 years ☐ 3-5 years ☐ > 5 years
**7**	Ultra-processed foods offered	☐ No<br>☐ Yes: …….% of total food assortment
**8**	Ready-to-eat foods offered	☐ No<br>☐ Yes

9	Food group	Examples	Whole or minimal processed	Ultra processed
1	CEREALS	bread, noodles, biscuits, cookies or any other foods made from millet, sorghum, maize, rice, wheat + insert local foods available grains, porridge or pastes or other locally available cereal foods	☐ Yes	☐ Yes
2	WHITE TUBERS AND ROOTS	white potatoes, white yams, cassava, or foods made from roots	☐ Yes	☐ Yes
3	VITAMIN A RICH VEGETABLES	pumpkin, squash, & other locally available vitamin-A rich vegetables, particularly that are orange inside (e.g. sweet pepper)	☐ Yes	☐ Yes
4	VITAMIN A RICH ROOTS AND TUBERS	carrots, sweet potatoes that are orange inside	☐ Yes	☐ Yes
5	DARK GREEN LEAFY VEGETABLES	dark green/leafy vegetables, including wild ones + *locally available vitamin-A rich leaves such as cassava leaves etc.*	☐ Yes	☐ Yes
6	OTHER VEGETABLES	other vegetables (e.g. tomato, onion, eggplant) , including wild vegetables	☐ Yes	☐ Yes
7	VITAMIN A RICH FRUITS	ripe mangoes, cantaloupe, dried apricots, dried peaches + other locally available vitamin A-rich fruits	☐ Yes	☐ Yes
8	OTHER FRUITS	other fruits, including wild fruits	☐ Yes	☐ Yes
9	ORGAN MEAT (IRONRICH)	liver, kidney, heart or other organ meats or blood-based foods	☐ Yes	☐ Yes
10	FLESH MEATS	beef, pork, lamb, goat, rabbit, wild game, chicken, duck, or other birds	☐ Yes	☐ Yes
11	FISH	fresh or dried fish or shellfish	☐ Yes	☐ Yes
12	INSECTS	termites, grass-hoppers, silk worm, larvae	☐ Yes	☐ Yes
13	EGGS	any eggs	☐ Yes	☐ Yes
14	MILK AND MILK PRODUCTS	milk, cheese, yoghurt or other milk products	☐ Yes	☐ Yes
15	NUTS AND SEEDS	nuts, seeds or foods made from these	☐ Yes	☐ Yes
16	LEGUMES	beans, peas, lentils or food made from these	☐ Yes	☐ Yes
17	OILS AND FATS	oil, fats or butter added to food	☐ Yes	☐ Yes

		or used for cooking		
18	SWEETS	sugar, honey, sweetened soda or sugary foods such as chocolates, sweets or candies	☐ Yes	☐ Yes
19	SPICES, CONDIMENTS, BEVERAGES	spices (black pepper, salt), condiments (soy sauce, hot sauce), coffee, tea, alcoholic beverages OR local examples		

Continue with Section D - questions Q11 and 12 - only for outlets that sell fresh vegetables, if not, STOP.

**10**	Shop level food safety claim (billboard/signboard OUTSIDE)	☐ No<br>☐ Yes	☐ Certification<br>☐ VietGAP<br>☐ PGS<br>☐ Other: …	☐ Informal claim:<br>☐ Rau An Toan<br>☐ Rau Sach<br>☐ Rau Huu Co<br>☐ Other: …	☐ Other communication<br>☐ Origin info<br>☐ Marketing health<br>☐ Marketing food safety
**11**	Fresh vegetable category food safety claim (billboard/signboard INSIDE the outlet for total category, around or on category shelf)	☐ No<br>☐ Yes	☐ Certification<br>☐ VietGAP<br>☐ PGS<br>☐ Other: …	☐ Informal claim:<br>☐ Rau An Toan<br>☐ Rau Sach<br>☐ Rau Huu Co<br>☐ Other: …	☐ Other communication<br>☐ Origin info<br>☐ Marketing health<br>☐ Marketing food safety

**12**	**Fresh Vegetables**	**Check box of foods offered**	**Food safety claim**		**List food safety claims/labels/brands observed (multiple answers possible)**
1	WHITE TUBERS AND ROOTS	☐ Potato<br>☐ Kohlrabi<br>☐ sweet potato (white)<br>☐ Other: …	☐ Yes all<br>☐ Some<br>☐ None	☐ Formal certification	☐ VietGAP<br>☐ PGS<br>☐ Other: …
2	VITAMIN A RICH VEGETABLES	☐ Pumpkin<br>☐ Red Capsicum/Bell pepper<br>☐ Other: …	☐ Yes all<br>☐ Some<br>☐ None	☐ Certification	☐ VietGAP<br>☐ PGS<br>☐ Other: …
				☐ Informal claim	☐ Rau An Toan<br>☐ Rau Sach

					☐ Rau Huu Co<br>☐ Other: …
				☐ Branding	☐ Retail brand<br>☐ Producer brand
				☐ Other communication	☐ Origin info<br>☐ Marketing on health<br>☐ Marketing on food safety
3	VITAMIN A RICH TUBERS	☐ Carrots<br>☐ Sweet potato (orange)<br>☐ Other: …	☐ Yes all<br>☐ Some<br>☐ None	☐ Certification	☐ VietGAP<br>☐ PGS<br>☐ Other: …
				☐ Informal claim	☐ Rau An Toan<br>☐ Rau Sach<br>☐ Rau Huu Co<br>☐ Other: …
				☐ Branding	☐ Retail brand<br>☐ Producer brand
				☐ Other communication	☐ Origin info<br>☐ Marketing on health<br>☐ Marketing on food safety
				☐ Informal claim	☐ Rau An Toan<br>☐ Rau Sach<br>☐ Rau Huu Co<br>☐ Other: …

				☐ Branding	☐ Retail brand<br>☐ Producer brand
				☐ Other communication	☐ Origin info<br>☐ Marketing on health<br>☐ Marketing on food safety
4	DARK GREEN LEAFY VEGETABLES	☐ Morning glory<br>☐ Cos Lettuce or dark green lettuce<br>☐Mustard leaves<br>☐ Pakchoy<br>☐Pumpkin leaves<br>☐Sweet potatoe leaves<br>☐ Spinach<br>☐Cai Lan (collards)<br>☐ Broccoli<br>☐ Other: (record) …	☐ Yes all<br>☐ Some<br>☐ None	☐ Certification	☐ VietGAP<br>☐ PGS<br>☐ Other: …
				☐ Informal claim	☐ Rau An Toan<br>☐ Rau Sach<br>☐ Rau Huu Co<br>☐ Other: …
				☐ Branding	☐ Retail brand<br>☐ Producer brand
				☐ Other communication	☐ Origin info<br>☐ Marketing on health<br>☐ Marketing on food safety
5	OTHER VEGETABLES	☐ Tomato<br>☐ Onion<br>☐ Eggplant<br>☐ Zucchini<br>☐ Corn<br>☐Green or yellow	☐ Yes all<br>☐ Some<br>☐ None	☐ Certification	☐ VietGAP<br>☐ PGS<br>☐ Other: …
				☐ Informal claim	☐ Rau An Toan<br>☐ Rau Sach<br>☐ Rau Huu Co

		capsicum<br>☐ Cauliflower<br>☐ White Cabbage Red/purple Cabbage<br>☐Light green lettuce<br>☐ Bamboo shoots<br>☐ Bitter Melon<br>☐Chayote<br>☐Cucumber<br>☐ Winter melon<br>☐ Bitter gourd<br>☐ Ceylon spinach<br>☐ Beans, list types: …<br>☐ Mushrooms, list types: …<br>☐ Other: (record) …			☐ Other: …
				☐ Branding	☐ Retail brand<br>☐ Producer brand
				☐ Other communication	☐ Origin info<br>☐ Marketing on health<br>☐ Marketing on food safety
