# Supplementary material for: Food safety and nutrition for low-income urbanites: exploring a social justice dilemma in consumption policy
Source: Reg Environ Change. 2019 Oct;31(2):397–420. doi: 10.1177/0956247819858019 (PMC7340485; doi:10.1177/0956247819858019)
Supplement: Supplementary file 3 [file EU-2019-0956247819858019-s3.pdf]

Food safety and nutrition for low-income urbanites:
exploring a social justice dilemma in consumption policy

Supplementary information

S3: HOUSEHOLD SURVEY QUESTIONNAIRE

<table>
<tr><td colspan="3">QC No:</td><td colspan="2">Interviewer No:</td></tr>
<tr><td colspan="3">Location/district:

Ba Dinh ……………………..1
Dong Da ……………………..2</td><td colspan="2">Day……………………Month……………. Year 2017

Monday ………………………………………1
Tuesday ………………………………………2
Wednesday …………………………………3
Thursday ……………………………………..4
Friday ………………………………………….5
Saturday ……………………………………..6
Sunday ………………………………………..7</td></tr>
<tr><td colspan="3">Respondent Name:</td><td colspan="2">Respondent phone No.:</td></tr>
<tr><td>Address:</td><td>GPS coordinates</td><td>No</td><td>Alley</td><td>Street/road</td></tr>
<tr><td colspan="3"><u>Questionnaire starting time:</u></td><td colspan="2"><u>Questionnaire finishing time:</u></td></tr>
</table>

INTERVIEWER'S STATEMENT	SUPERVISOR'S STATEMENT
I have finished the questionnaire according to the instructions of the project<br><br>(Signature and name in full)	I have checked this questionnaire according to the instructions of the project<br><br>(Signature and name in full)

[SA] = Single Answer only | [MA] = Multiple Answers possible/allowed |[OA] = Open Answer

Include WOMEN only

Include only the respondents that are willing to participate in both this survey AND the nutrition survey

Part 1- Screening

Q1	Please answer the following question.<br>Who is the main person responsible for the food shopping in your household? [SA]	Code	Route
	I am	1	Continue
	Other person	2	Ask to talk to this other person if not possible or if not female stop

Q2	Please note down the gender of the respondent [SA]	Code	Route
	Female	1	Continue
	Male	2	Stop

Q3	Would you be willing to also participate in an additional research about the foods consumed in your household? [for explanation please refer to the informed consent information above]	Code	Route
	Yes	1	Continue

	No	2	Stop

Q4	Who does the food preparation/cooking in your household? [SA]	Code	Route
	**I am the ONLY** person in charge of deciding on the menu of the day, the preparation of the ingredients and the cooking of the dishes.	1	Continue
	There is no clear division of labour. We are all in charge of deciding on the menu of the day, the preparation of the ingredients and the cooking of the dishes.	2	
	Someone else / some others are in charge of deciding on the menu of the day, the preparation of the ingredients and the cooking of the dishes.	3	Stop

Q5	What is your year of birth? [OA]		
	…………………………………………………………………………………………………		
	During or after 1967	1	Continue
	Before 1967	2	Stop

Q6	How long have you been living here at this address? [SA]	Code	Route
	Less than 2 years	1	Stop
	2 years or more	2	Continue

Q7	What is your household size? [SA]	Code	Route
	Single household	1	Stop
	2 or more people	2	Continue

Q8	A	How many persons are living within your household? [SA]							
			2 pers	3 pers	4 pers	5 pers	6 pers	7 pers	8 pers	8+ pers
B		Code	1	2	3	4	5	6	7	8
What is your households monthly income	Less than 3 mln vnd	1	Continue							
	3,000,000 - 4,499,999	2								
	4,500,000-5,499,999	3								
	5,500,000-6,499,999	4								
	6,500,000-7,499,999	5	STOP	Continue						
	7,500,000-8,499,999	6	STOP							
	8,500,000-9,499,999	7	STOP							
	9,500,000-10,499,999	8	STOP							
	10,500,000-11,499,999	9	STOP	STOP	Continue					

	11,500,000-12,499,999	10	STOP	STOP		
	12,500,000-13,499,999	11	STOP	STOP		
	13,500,000-14,999,999	12	STOP	STOP	STOP	Continue
	15mln and over	13	STOP			

Part 2- Food shopping PRACTICES

Q9	How many times per week do you/your family buy food for your household? [SA]	Code
	Every day	1
	4-6 times a week	2
	2 to 3 times a week	3
	Once a week	4

Q10	How often do you buy your food at these places? [SA – Showcard outlets]					
		Hypermarket/ Supermarket	Formal wet market	Informal street market	Convenience store/ Minimart	Specialty store	Street vendor
	Every day	1	1	1	1	1	1
	4-6 times a week	2	2	2	2	2	2
	2-3 times a week	3	3	3	3	3	3
	Once a week	4	4	4	4	4	4
	< once a week	5	5	5	5	5	5
	Never	6	6	6	6	6	6

Q11	Could you please tell me the name of the supermarket and/or official wet market you where you shop at least once a week? [OA]<br>If no hyper/supermarket or wet market is visited once per week in Q10, please continue with Q12		
		Code	Name of the place/address
	Hypermarket/ Supermarket	1	
	Formal Wet market	2	

Q12	At what moment of the day do you normally shop? [SA]	Code
	Early morning: before 8:00	1
	Morning: 8:00-11:00	2
	Lunch: 11:00-13:00	3
	Afternoon: 13:00-16:30	4
	Late afternoon: 16:30-19:00	5
	Evening: after 19:00	6

Q13	Where (what type of outlet) do you/your household buy most (QUANTITY) of the foods your household consumes during a week? [SA – Showcard outlets]	Code
	Hypermarket/ Supermarket	1
	Formal wet market	2
	Informal street market	3
	Convenience store/ Minimart	4
	Specialty store (e.g. safe vegetable shop, fruits shop, meat shop,…)	5
	Street vendor	6

Q14	How much do you agree with the following statements? [SA per statement]					
	"**The reason I buy most food from** the shop I buy most of my foods (place is the highest ranking answer Q10a) **is because (of)**…"	Totally disagree	Disagree	Neutral	Agree	Totally agree
A	I am used to shop here; it's a habit	1	2	3	4	5
B	Lowest selling price	1	2	3	4	5
C	It is close to my home	1	2	3	4	5
D	It is located on the way from home to work/school/…	1	2	3	4	5
E	The foods offered are safe for consumption	1	2	3	4	5
F	I enjoy shopping here	1	2	3	4	5
G	Wide assortment offered	1	2	3	4	5
H	They offer healthy foods	1	2	3	4	5
I	The personal contact with the vendor	1	2	3	4	5

Q15	What means of transportation do you use for shopping foods for your household? [MA possible and please indicate the frequency of transportation means]					
		Never	Seldom	Sometimes	Most of	Always

					the times	
A	None, I walk	1	2	3	4	5
B	Bicycle	1	2	3	4	5
C	Motorbike	1	2	3	4	5
D	Car	1	2	3	4	5

Part 3- Food shopping PREFERENCES

Q16	How many times per week do you/your family PREFER to shop for food for your household? [SA]	Code
	Every day	1
	4-6 times a week	2
	2 to 3 times a week	3
	Once a week	4

Q17	At what moment of the day would you PREFER to shop? [SA]	Code
	Early morning, before 8:00	1
	Morning between 8:00-11:00	2
	Lunch: between 11:00-13:00	3
	Afternoon between 13:00-16:30	4
	Late afternoon: 16:30-19:00	5
	Evening: after 19:00	6

Q18	Where (what type of outlet) would you PREFER to buy food for your household? [SA – Showcard outlets]	Code
	Hypermarket/ Supermarket	1
	Official wet market	2
	Informal street market	3
	Convenience store/ Minimart	4
	Specialty store	5
	Street vendor	6

Q19	How much do you agree with the following statements? [SA per statement]					
		Totally disagree	Disagree	Neutral	Agree	Totally agree
A	Buying food in supermarkets is too time consuming	1	2	3	4	5
B	Buying food in supermarkets is	1	2	3	4	5

	interesting					
C	Buying food in supermarkets is desirable	1	2	3	4	5
D	Buying food in supermarkets is expensive	1	2	3	4	5
E	Foods offered in supermarkets are safe for consumption	1	2	3	4	5
F	If I could I would only buy at supermarkets	1	2	3	4	5
G	Buying food at wet markets is too time consuming	1	2	3	4	5
H	Buying food at wet markets is interesting	1	2	3	4	5
I	Buying food at wet markets is desirable	1	2	3	4	5
J	Buying food at wet markets is expensive	1	2	3	4	5
K	Foods offered at wet markets are safe for consumption	1	2	3	4	5
L	If I could I would only buy at wet markets	1	2	3	4	5

Part 4- Food selection

Q20	What is the average daily food budget of your household? [SA]	Code
	Less than 50,000 VND	1
	50,000- 99,999 VND	2
	100,000-199,999 VND	3
	200,000VND-299,999 VND	4
	300,000-400,000 VND	5
	More than 400,000 VND	6

Q21	Where do you usually buy the following food items? [SA – Showcard outlet types]]						
		Hyper-/ Superm arket	Formal wet market	Informal Street market	Conveni ence store	Specialt y store	Street vendor
A	Rice, cereals and starch products	1	2	3	4	5	6
B	Fish and Seafood	1	2	3	4	5	6
C	Meat, chicken (including organ)	1	2	3	4	5	6

D	Eggs	1	2	3	4	5	6
E	Dark green leafy vegetables	1	2	3	4	5	6
F	Roots and tubers	1	2	3	4	5	6
G	Orange vegetables (pumpkin, sweet potato, carrot, red bell pepper)	1	2	3	4	5	6
H	Orange flesh fruits (ripe papaya, ripe mango, persimmon, passionfruit, orange muskmelon/rockmelon, dried apricots or peaches, NOT: Orange fruit (qua cam))	1	2	3	4	5	6
I	Other fruits and vegetables	1	2	3	4	5	6
J	Legumes	1	2	3	4	5	6
K	Dairy products	1	2	3	4	5	6
L	Snack foods, ultra-processed foods (e.g. instant noodles)	1	2	3	4	5	6
M	Spices, condiments, beverages	1	2	3	4	5	6
N	Oils and fats	1	2	3	4	5	6
O	Nuts and seeds	1	2	3	4	5	6
P	Insects	1	2	3	4	5	6
Q	Sweets	1	2	3	4	5	6

Q22	Which food items would you prefer to buy **more** if your food budget increases? [Top 3]	Code
	Rice, cereals and starch products	1
	Fish and Seafood	2
	Meat, chicken (including organ)	3
	Eggs	4
	Dark green leafy vegetables	5
	Roots and tubers	6
	Orange vegetables (pumpkin, sweet potato, carrot)	7
	Orange flesh fruits (papaya, mango)	8
	Other fruits and vegetables	9

	Legumes	10
	Dairy products	11
	Snack foods, ultra-processed foods (e.g. instant noodles)	12
	Spices, condiments, beverages	13
	Oils and fats	14
	Nuts and seeds	15
	Insects	16
	Sweets	17

Q23	How important are the following factors to you when you decide what vegetables to buy? [SA]					
		Totally unimporta nt	Unimportan t	Neutral	Importan t	Absolutely important
A	Taste preference of me and my family members	1	2	3	4	5
C	Food safety	1	2	3	4	5
B	Health (nutritional value)	1	2	3	4	5
D	Easy to prepare	1	2	3	4	5
E	Low price	1	2	3	4	5

Q24	Please finish the following sentence and indicate the importance of the complete statement. *'It is important to me that the food I eat on a typical day.....'*					
		Totally unimporta nt	Unimport ant	Neutral	Importan t	Absolutely important
A	Contains a lot of vitamins and minerals	1	2	3	4	5
B	Keeps me healthy	1	2	3	4	5
C	Is nutritious	1	2	3	4	5
D	Is high in protein	1	2	3	4	5
E	Is easy to clean	1	2	3	4	5
F	Can be prepared easily	1	2	3	4	5
G	Can be bought close to where I live or work	1	2	3	4	5
H	Is easily available	1	2	3	4	5
I	Is affordable	1	2	3	4	5
J	Is cheap	1	2	3	4	5
K	Is good value for money	1	2	3	4	5

Part 5 – VEGETABLES - Food safety RISK perception

Q25	How much do you agree with the below statement regarding your concern with the safety of the vegetables you buy for your household? [SA]					
		Totally not agree	Not agree	Neutral	Agree	Totally Agree
A	I am ALWAYS concerned (frequency)	1	2	3	4	5
B	I am VERY MUCH concerned (severity)	1	2	3	4	5

Q26	How concerned are you about the following sources of food safety risks[SA]					
		Totally not concerned	Not so concerned	Neutral	Rather concerne d	Totally concerned
A	Fertilizers and growth enhancers	1	2	3	4	5
B	Pesticides	1	2	3	4	5
C	Preservation treatment	1	2	3	4	5
D	Use of /growing with contaminated water and soil	1	2	3	4	5
E	Unhygienic handling practices (farm to store)	1	2	3	4	5

Q27	How much do you agree with the following statements?					
		Totally disagree	Disagr ee	Neutral	Agree	Totally Agree
A	I only select vegetables that have a peel	1	2	3	4	5
B	I only select vegetables which look fresh/green	1	2	3	4	5
C	I only select vegetables which are a bit eaten by worms	1	2	3	4	5
D	I only select vegetables that are on season	1	2	3	4	5
E	I only select vegetables that carry food safety certification	1	2	3	4	5
F	I only buy vegetables when I know where they are produced	1	2	3	4	5
G	I buy what my vendor advices me to buy	1	2	3	4	5

Q28	How much do you agree with the following statements? [SA]					
		Totally disagree	Disagree	Neutral	Agree	Totally agree
A	I am less concerned about the safety of vegetables when they are in-season than off-season.	1	2	3	4	5
B	Vegetables offered in supermarkets are safer than in wet markets.	1	2	3	4	5
C	Vegetables offered in wet markets are safer than in street markets/vendors	1	2	3	4	5
D	It is safer to consume vegetables that are certified safe than uncertified vegetables.	1	2	3	4	5
E	Vegetables offered in wet markets are safer than in supermarkets	1	2	3	4	5
F	Vegetables offered in street markets/vendors are safer than in wet markets	1	2	3	4	5
G	I feel more safe when I buy from a vendor I trust, than when I buy certified vegetables	1	2	3	4	5
H	I am concerned about safety but make most purchasing decisions based on price	1	2	3	4	5

Q29	If you are shopping for vegetables, but are worried about food safety in the retail outlet, what do you do? [SA]<br><br>Retail outlet refers to the place where you are shopping for vegetables; [Showcard of retail types can be used if unclear to the respondent]	Code
	Buy the same food and quantity anyway	1
	Buy the same food but a lesser quantity	2
	Buy different/less preferred food	3
	Change retail outlet	4

Q30	How often do you feel confident in protecting yourself and your household members from food safety risks? [SA]	Code
	Yes, always	1
	Yes, sometimes	2
	Neutral	3
	Seldom	4
	Never	5

Q31	Independently from the source, can you rate how you generally consider the following vegetables in terms of safety? (1 would mean "Not safe at all" and 5 would mean "Very safe" with each statement)					
		Not safe at all	Risky	Neutral	Rather safe	Very safe
A	Roots and tubers	1	2	3	4	5
B	Squash, gourds, pumpkin	1	2	3	4	5
C	Dark green leafy vegetables	1	2	3	4	5
E	Orange sweet potato	1	2	3	4	5
F	Carrots	1	2	3	4	5
G	Beans and legumes	1	2	3	4	5
H	Tomato	1	2	3	4	5
I	Red capsicum	1	2	3	4	5
J	Green beans	1	2	3	4	5
K	Cabbage	1	2	3	4	5
L	Mushrooms	1	2	3	4	5
M	Kang kong/ morning glory	1	2	3	4	5
N	Light green lettuce	1	2	3	4	5
O	Cauliflower, broccoli	1	2	3	4	5
P	Pak choy					
Q	Zucchini	1	2	3	4	5
R	Eggplant	1	2	3	4	5

Part 6 – VEGETABLES – TRUST in Food Safety

Q32	To what extent do you believe that the vegetables sold under below mentioned claims are safe? [Showcard food safety claim indicators]					
		I totally	I do not	neutral	I moderately	I totally

		NOT believe	believe		believe	believe
A	VietGAP certification	1	2	3	4	5
B	Rau an toan (safe vegetables)	1	2	3	4	5
C	Rau sach (clean vegetables)	1	2	3	4	5
D	Rau Huu Co (organic vegetables)	1	2	3	4	5
E	Private label (retail brand)					
F	Producer brand					

Q33	What makes you trust the safety of the vegetables the most? [SA]	Code
	Your own judgement based on the external appearance of vegetables	1
	Certificate given by authority attesting that the vegetable is safe	2
	Retailer branding	3<br>3
	Advice from regular vendor about food safety	4
	Advice from relatives/friends/neighbours	5
	Information on producer and production area	6
	Others, please specify……….	7

Q34	Do you think the way you clean vegetables is enough to make them safe for consumption?	Code
	Yes, totally	1
	Yes, partly	2
	No, not at all	3

Q35	Have you or your family ever been sick due to consumption of vegetables?	Code
	Yes	1
	No	2

Q36	The following statements are about problems that you might face when buying **<u>SAFE</u>** food. How much do you agree with the following statement? (Single code)					
	**"When I want to buy <u>SAFE</u> food,…."**	Totally disagree	Disagree	Neutral	Agree	Totally agree

A	the price is too high.	1	2	3	4	5
B	they are often not available.	1	2	3	4	5
C	they are often not fresh.	1	2	3	4	5
D	the assortment is limited.	1	2	3	4	5
E	I don't trust the food safety claim	1	2	3	4	5
F	there are no problems for me.	1	2	3	4	5

Part 7 – Respondent's information

Q37	What is your highest education level? [SA]	Code
	No formal education	1
	Primary school	2
	Secondary school	3
	High school	4
	Vocational school	5
	Undergraduate	6
	Postgraduate	7

Q38	Do you care for …. within your household? [MA possible]		
		Code	
		Yes	No
A	Children 0-4 yrs	1	2
B	Children 5-12yrs	1	2
C	Elderly people (60yrs and older)	1	2
D	People that are ill	1	2

Q39	Is a woman from an older generation living with you within the household, who is or was responsible for food shopping? [SA]	Code	Route
	Yes	1	Continue with Q40
	NO	2	STOP

Q40	Would you be willing to participate in a follow-up more in-depth research later this year/next year, which includes a multi- generation household interview? [SA]	Code

	Yes	1
	No	2

END
