# Supplementary material for: Food safety and nutrition for low-income urbanites: exploring a social justice dilemma in consumption policy
Source: Reg Environ Change. 2019 Oct;31(2):397–420. doi: 10.1177/0956247819858019 (PMC7340485; doi:10.1177/0956247819858019)
Supplement: Supplementary file 4 [file EU-2019-0956247819858019-s4.pdf]

Food safety and nutrition for low-income urbanites:
exploring a social justice dilemma in consumption policy

Supplementary information

S4: ANALYSIS FRAMEWORK

Combine dataset:	With dataset:	Analyses
Census	Household survey	- Where, when, how (transport means) and what people buy related to food retail proximity and available assortment<br>- Where and what people buy related to risk perception and trust
24-hour dietary recall	Census	- What people eat and where it is purchased in relation to food retail availability
24-hour dietary recall	Household survey	- What people eat and where it is purchased in relation to food shopping practices and preferences<br>- What people eat and where it is purchased in relation to food risk perception and trust (vegetables only)
Price data (vegetables)	Census<br>Household survey<br>24-hour dietary recall	- Where and what people buy related to food retail availability AND prices<br>- Where and what people buy related to risk perception, trust AND prices
